# Scaling the Feeding Mechanism of Captive *Alligator mississippiensis* from Hatchling to Juvenile

**DOI:** 10.3390/biology3040724

**Published:** 2014-11-10

**Authors:** James R. Kerfoot, Micah P. Fern, Ruth M. Elsey

**Affiliations:** 1 Department of Biological Sciences, Union University, 1050 Union University Drive, Jackson, TN 38305, USA; E-Mail: micahfern@gmail.com; 2 United State Department of Agriculture, Auburn University, 602 Duncan Drive, Auburn, AL 36849, USA; 3 Louisiana Department of Wildlife and Fisheries, Rockefeller Wildlife Refuge, 5476 Grand Chenier Hwy, Grand Chenier, LA 76043, USA; E-Mail: relsey@wlf.la.gov

**Keywords:** American alligator, ontogenetic niche shift, prey-capture kinematics

## Abstract

Small changes in size can lead to potential performance consequences and may influence an organism’s ability to utilize resources in its environment. As the American alligator (*Alligator mississippiensis*) transitions between neonate, juvenile and adult habitats (ontogenetic niche shifts), there are inevitably dynamic changes in their feeding performance. This study sought to investigate the scaling of the feeding mechanism and its performance from hatchling to juvenile size classes in *A. mississippiensis*. Feeding events were recorded during March 2011 at Rockefeller Wildlife Refuge (Grand Chenier, Louisiana). Thirty-six captive individuals were randomly sampled, ranging from 30.5 cm to 91.5 cm total length, and feeding events were recorded using a high speed camera at a rate of 300 fps. Results indicated that many linear, angular and timing kinematic variables scale allometrically with cranium length; whereas maximum gape velocity and duration of feeding bout do not scale with cranium length and remain constant between these size classes. Although it has been shown that there is an isometric relationship between cranial elements and body size in *A. mississippiensis*, this relationship is not transferred to linear and timing variables of prey-capture events. These allometric relationships echo other investigations of scaling relationships such as bite-force production and terrestrial locomotion.

## 1. Introduction

Small changes in morphology or simple changes in size can lead to novel functions and the potential for performance consequences [1]. An organism’s ability to utilize its environment is often determined by body size and its influence on performance [2]. Weighing approximately 65 g at birth and eventually growing 4000-fold by adulthood, *Alligator mississippiensis* (Daudin 1801) provides a model to investigate the influence of body size on performance [3,4]. Available data support the inference that as *A. mississippiensis* grow, they experience ontogenetic niche shifts, with a strong decline in terrestrial performance (i.e., speed, frequency and duration of terrestrial locomotion) [3,5,6,7]. Throughout ontogeny limbs become relatively shorter, with distal limb segments becoming increasingly shorter relative to proximal segments [3,7,8]. This negative allometry of distal limb lengths and proportions causes *A. mississippiensis* to become more dependent on aquatic environments as growth increases [3]. Conversely, another study found that limb muscle mass scaled isometrically with body mass, suggesting that limbs potentially undergo a corresponding increase in girth [7].

With the transition from terrestrial to more aquatic environments, and the significant change in body size ontogenetically, the feeding apparatus inevitably changes as well. Ontogenetic changes have been observed in the feeding apparatus where hatchlings tend to have short, broad snouts that transition to elongate, slender snouts in adults [3,4,9,10]. These ontogenetic changes in the feeding apparatus are thought to correlate with shifts in diet. Initially diet is composed of insects and small fish moving to incorporate large mammals, reptiles and birds in adulthood [4,5,11,12,13]. However, bite force has been shown to scale in a positive allometric relationship with respect to body mass, total length and cranium length and did not vary in association with major shifts in diet [4,9].

While terrestrial locomotion may decline as a result of increasing body size and niche shifts, how do the feeding apparatus and associated performance parameters fare? Although there is a noted decline in terrestrial locomotion, and crocodilians must utilize the feeding apparatus throughout ontogeny, little is known about the scaling of the feeding mechanism. How does the anatomical scaling of the cranial elements influence the physiological scaling of prey capture? In an attempt to establish the functional changes that occur in the feeding mechanism as a consequence of body size, this study was designed to investigate the scaling of prey capture in hatchling/juvenile *A. mississippiensis*, ranging from 30.5 cm to 91.5 cm in total length (TL). The primary objectives of this study were to: (1) identify the scaling relationship between the cranial elements and body length; (2) describe the scaling relationships between cranium length and prey capture kinematic variables; and (3) compare the observed scaling relationships of prey capture events to a model that assumes isometry in prey capture events, the geometric similarity model (GSM). While this is not the first study to investigate the kinematics of prey capture in crocodilians [4,14,15], it is the first to investigate the scaling relationships of linear, angular and timing excursions of the feeding mechanism in hatchlings and juveniles. It was hypothesized that observed scaling of the kinematic variables are not significantly different from the scaling proposed by the geometric similarity model (GSM).

## 2. Experimental Section

While the use of captive-raised individuals are commonly utilized in performance studies [16], concern with their use and inference to natural, wild-type counterparts has been debated [16,17]. However, it has been shown that there are documented anatomical differences between captive and wild *A. mississippiensis* [17] that may have influences on the performance parameters measured here, warranting caution when extrapolating implications from this study to wild individuals.

### 2.1. Scaling of the Cranial Elements

**Figure 1:**
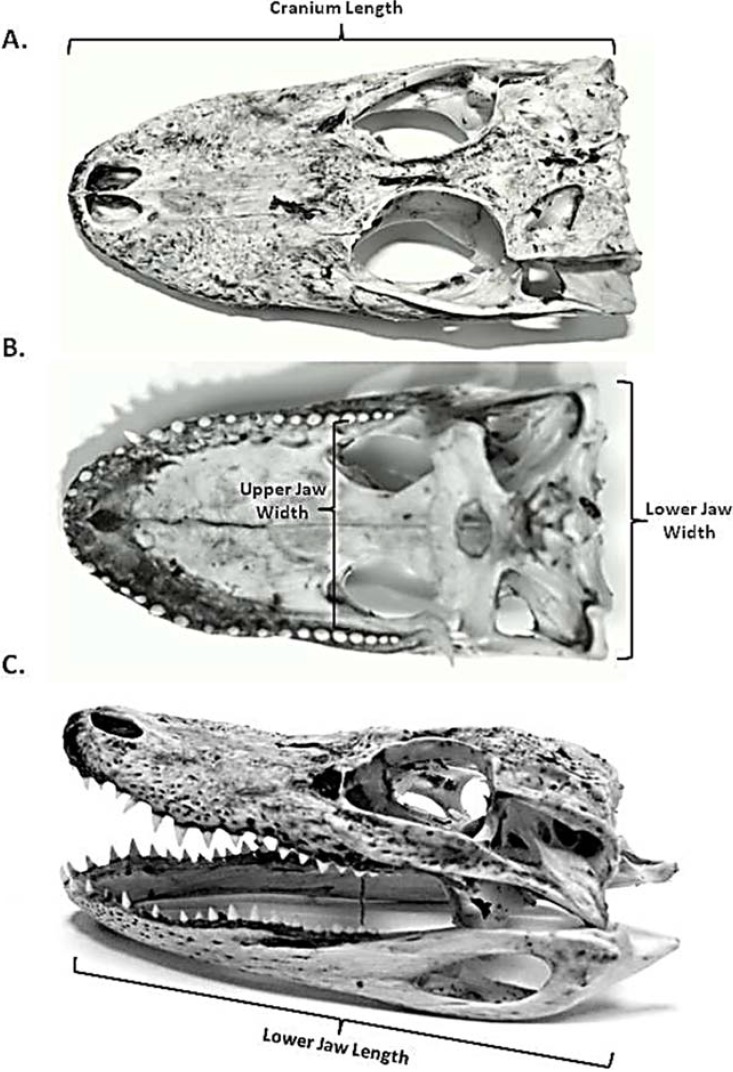
Diagram depicting the cranial measurements used to describe the relationship between cranial elements of the feeding mechanism and standard length. (**A**) Dorsal view of neurocranium. Cranial length was defined as the distance between the posterior margin of the parietal bone and the anterior margin of the premaxilla; (**B**) Palatal view of the neurocranium. Lower jaw width was defined as the distance between the articular condyles; upper jaw width was defined as the shortest distance between the lateral margins of the maxilla; (**C**) Left lateral view of the neurocranium and mandible. Lower jaw length was defined as the distance between the articular-quadrate joint to the anterior margin of the dentary bone.

To establish the scaling relationships between the cranial elements and estimated total length (from the tip of snout to the tip of the tail); *A. mississippiensis* skulls were obtained from Brooks Brothers Alligator Farm located in Christmas, Florida and from the Rockefeller Wildlife Refuge in Grand Chenier, Louisiana (seven and nine skulls, respectively, *n* = 16). Size information sent with the skulls established six size classes ranging from 30.5 cm to 274.3 cm in TL (Table 1). Four cranial elements were measured: cranium length, lower jaw length, lower jaw width and upper jaw width (Figure 1). Cranium length was defined as the distance between the posterior margin of the parietal bone and the anterior margin of the premaxilla. Lower jaw length was measured from the posterior surface of the articular-quadrate joint to the anterior margin sof the dentary bone. Lower jaw width was measured as the distance between the lateral surfaces of the condyles. Upper jaw width was measured between the lateral margins of the maxilla (in the palatal view) at the anterior edge of the posterior palatine vacuities. Data were log-transformed and a linear regression was utilized to establish the scaling relationship between estimated total length and the cranial elements. To control Type I error rates due to multiple measurements on the same individual a Bonferroni corrected α-value was set at 0.0125 for this section of the study (0.05/4 = 0.0125) [18].

**Table 1:** Mean and variation of cranial elements measured for *A. mississippiensis*.

Estimated Size Class (cm)		Cranium Length (cm)	Lower Jaw Length (cm)	Lower Jaw Width (cm)	Upper Jaw Width (cm)
	n	Average ± SE	Average ± SE	Average ± SE	Average ± SE
30.5–60.5	3	5.5 ± 1.04	5.6 ± 1.04	2.1 ± 1.05	2.1 ± 1.02
60.5–91.5	3	7.4 ± 1.01	8.1 ± 1.03	2.3 ± 1.04	3.1 ± 1.02
91.5–122.0	3	9.8 ± 1.08	11.0 ± 1.07	3.4 ± 1.07	4.1 ± 1.05
152.0–182.6	2	21.4 ± 0.00	24.5 ± 1.04	7.9 ± 1.11	8.1 ± 0.00
213.0–244.0	3	33.1 ± 1.04	40.7 ± 1.04	13.2 ± 1.03	13.5 ± 1.03
244.0–274.3	2	40.7 ± 1.02	50.1 ± 1.02	16.2 ± 1.05	16.6 ± 1.04

### 2.2. Kinematic Scaling of the Feeding Mechanism

**Figure 2:**
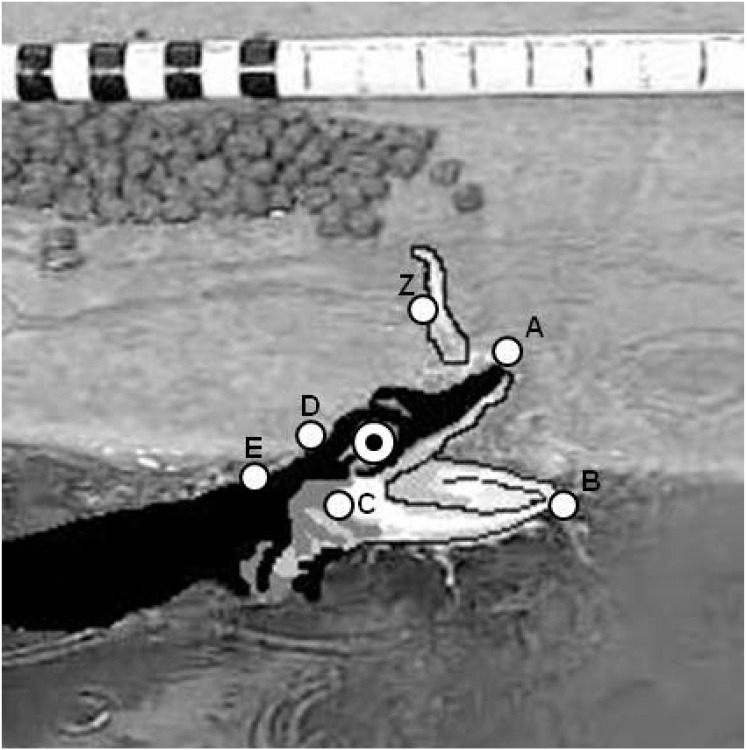
Diagram of *A. mississippiensis* portraying landmarks used to measure linear excursions and angular displacements during a feeding event. (**A**), anterior tip of the premaxilla; (**B**), anterior tip of the dentary bone; (**C**), articular-quadrate joint; (**D**), posterior margin of the parietal bone; E, base of the neck/superior margin of the scapula; Z, morsel of food used in feeding event.

Feeding events of captive *A. mississippiensis* were recorded on 21 March 2011 at Rockefeller Wildlife Refuge (Grand Chenier, Louisiana). During the normally scheduled feeding time, 36 individuals were randomly sampled from holding tanks, ranging from 30.5 cm to 91.5 cm TL (spanning hatchling and juvenile sizes, [5,13,19]). Individuals were not fed for 3–4 days prior to the recording of the feeding events. Feeding events were recorded over a three hour period between 1000 hours and 1300 hours using a Casio Exilim-FX high speed video camera at a rate of 300 fps. The camera was mounted on a tripod stand at a height of approximately 1.4 m over the holding tanks. Strips of raw chicken were suspended by monofilament line 3 cm in front of the individual. Only the first feeding events and those that occurred perpendicular to the camera viewing frame were utilized for video analysis (Figure 2). A meter stick was placed in the filming tank to set the scale when analyzing the video.

Eight linear, angular, timing and velocity variables were measured from each video clip using Coach 6 Studio MV Software (Center for Microcomputer Applications, Netherlands). The kinematic variables measured were (Table 2, Figure 2): maximum gape (mm), cranial rotation (degree), lower jaw displacement (degree), time to maximum gape, duration of feeding bout, maximum gape velocity, lower jaw displacement velocity, and cranial rotation velocity. Linear and angular measurements were calculated as the greatest excursion of the variable from a resting, mouth-closed position. Velocity and timing variables were measured from time zero (t_0_) which was defined as the frame prior to the mouth opening. Data were log-transformed and a series of linear regressions were utilized to establish the scaling relationship between estimated cranium length and the kinematic variables. To control Type I error rates due to multiple measurements on the same individual a Bonferroni corrected *p*-value of 0.006 (0.05/8 variables = 0.006) was utilized in these analyses.

**Table 2:** Linear excursion, angular displacement and timing variables quantified in this study.

Variable	Units	Definition
Cranium Length	mm	Length from the anterior tip of the premaxilla (A) to the posterior portion of the parietal bone (D). See Figure 2.
Maximum Gape	mm	Maximum distance from the anterior tip of the premaxilla (A) to the anterior tip of the dentary bone (B). See Figure 2.
Lower Jaw Displacement	degree	Maximum postero-ventral rotation of the lower jaw relative to the neurocranium, measured by the angle formed by line segments AC and BC. See Figure 2.
Cranial Rotation	degree	Maximum postero-dorsal rotation of the neurocranium relative to the body, measured by the angle formed by line segments AD and DE. See Figure 2.
Time Zero (t₀)		The frame prior to mouth opening.
Time to Maximum Gape	s	Time measured from t₀ until maximum gape.
Duration of Feeding Bout	s	Times measured from t₀ until individual fully closed its mouth.
Maximum Gape Velocity	mm s^−1^	Velocity measured from t_0_ until maximum gape.
Lower Jaw Displacement Velocity	degree s^−1^	Velocity measured from t_0_ until maximum displacement.
Cranial Rotation Velocity	degree s^−1^	Velocity measured from t_0_ until maximum rotation.

### 2.3. Geometric Similarity Model

To test the hypothesis that observed scaling of the kinematic variables are not significantly different from the expected scaling proposed by a mechanical model, a geometric similarity model (GSM) depicting the expected scaling relationships for increasing body size and feeding kinematics in toads was employed (following [2,20]). The GSM states that the cross-sectional area of muscle and body mass would be expected to increase by the second and the third power of body length, respectively [2]. This predicts that as body size increases, force production of the muscle units powering the movements would increase at a slower rate than the mass of components being moved [2]. The geometric similarity model would predict that linear and angular velocities would scale with a slope of 0 and -1, respectively, when log-transformed and plotted against the log-transformation of cranium length (or equally with total length as this study indicates) [2]. Linear and angular displacements would scale with a slope of 1 and 0, respectively; and timing variables would scale with a slope of 1 (following [2,20,21]). The expected scaling relationships from the GSM were used as a basis for comparing the observed scaling relationships for the kinematic variables (following [2]).

To compare the observed scaling relationships with those predicted from the GSM a series of student *t*-tests were employed to compare the observed least square slopes to those generated from the predicted model. For testing the scaling of cranial elements against GSM, a Bonferroni corrected *p*-value of 0.0125 (0.05/4 variables = 0.0125), and for the comparison of the GSM to the kinematic variables a Bonferroni corrected *p*-value of 0.006 was employed.

### 2.4. Statistics

To investigate the scaling relationship between body size and prey-capture kinematics, the measured variables were log-transformed and regressed against the log-transformed cranium length, using a series of linear regressions. Variables were regressed against cranium length because the lengths of each individual could not be measured from the videos. All statistical analyses were performed in SPSS Statistics 19.0 (IBM Inc., Armonk, NY, USA) and Sigma Plot 2001 (Systat Software Inc., San Jose, CA, USA) with the appropriate Bonferroni corrected *p*-values indicated above.

## 3. Results and Discussion

### 3.1. Scaling of the Cranial Elements

**Figure 3:**
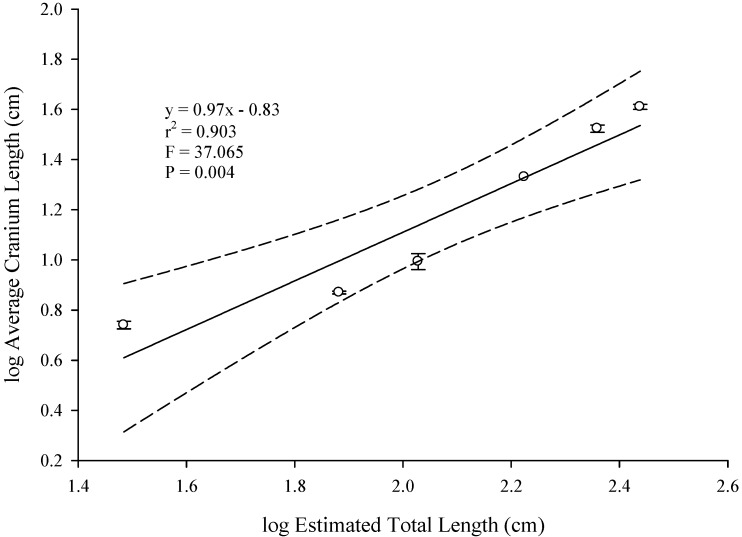
Relationship between estimated total length and cranium length in *A. mississippiensis*. Linear regression is represented by a solid line (—) and the 95% confidence intervals (upper and lower bounds) are represented by dashed lines (– – –).

**Figure 4:**
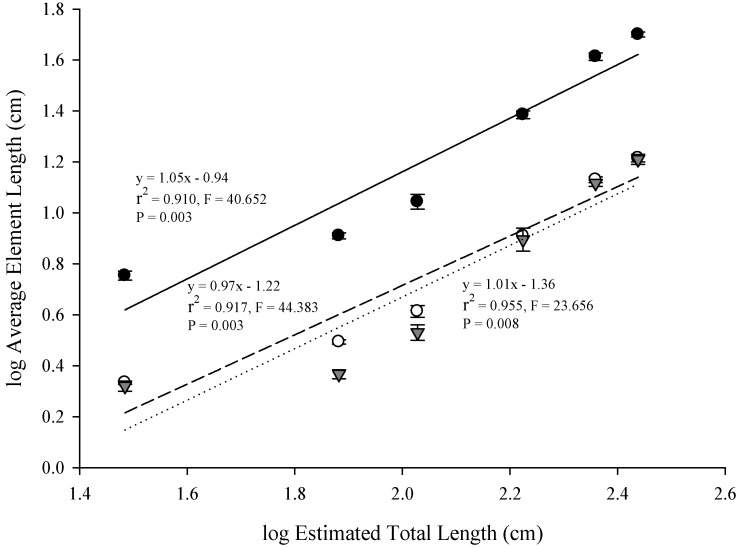
Relationships between estimated total length and the cranial element lengths in *A. mississippiensis* skulls. Lower jaw lengths are represented by closed circles (●), upper jaw widths by open circles (○) and lower jaw widths by closed triangles (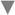). The solid regression line represents the relationship between total length and lower jaw length, dashed line between total length and upper jaw width and the dotted line between total length and lower jaw width.

The mean log-transformed estimates of the cranial elements in *A. mississippiensis* increase as size classes increase (Table 1). Average cranium length scaled significantly with positive isometry against estimated average log total length; maintaining geometric similarity from hatchling throughout juvenile sizes (slope = 0.97,* r*^2^ = 0.903, *p* = 0.004, Figure 3, Table 3). This indicated that log cranium length is isometric across size classes. Investigating the scaling of the jaw elements further, log lower jaw length, log upper jaw width and log lower jaw width scaled isometrically with average log total length in *A. mississippiensis* (Figure 4). Regression slopes for these relationships ranged from 0.97 to 1.05, indicating the maintenance of geometric similarity as individuals increased in total length. Results of the series of *t*-tests comparing the observed cranial element slopes against those predicted by the GSM indicated no significant difference between them (all *p*-values > 0.200, Table 3). These analyses validate the use of cranium length as a proxy for body size in subsequent analyses on the scaling relationships in the kinematic variables.

### 3.2. Kinematic Scaling of the Feeding Mechanism

**Figure 5:**
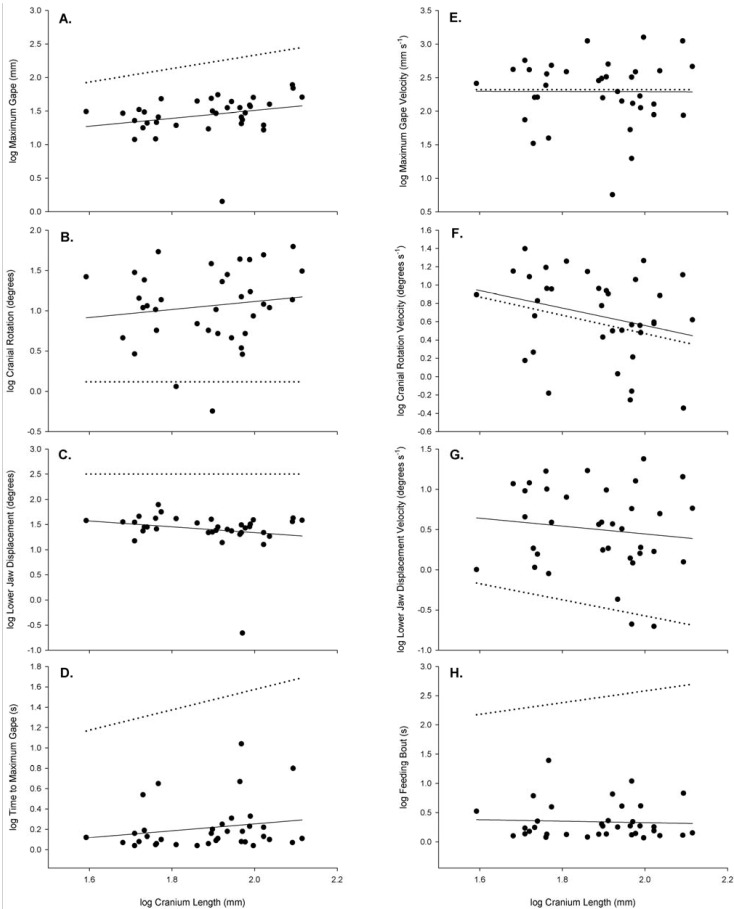
Relationships between cranium length and the feeding kinematic variables measured: (**A**) maximum gape; (**B**) cranial rotation; (**C**) lower jaw displacement; (**D**) time to maximum gape; (**E**) maximum gape velocity; (**F**) cranial rotation velocity; (**G**) lower jaw displacement velocity and (**H**) feeding bout. All variables were log-transformed for the analysis. Solid lines represent observed regression lines and dotted lines are expected regression lines generated by the geometric similarity models.

There was large variability in the datasets when investigating the relationships between log transformed cranium length and the kinematic variables (Figure 5, Table 3). Maximum gape and cranial rotation had a positive trend with cranium length, although both were non-significant as indicated by the very low coefficients of determination (0.071 and 0.020, respectively, Table 3). Maximum gape and cranial rotation increased with size at a slower rate than dictated by the GSM as indicated by the observed least squares slopes (Table 3, Figure 5A,B). Both observed least squares slopes for maximum gape and cranial rotation were significantly different from slopes predicted by the GSM (both *p*-values < 0.0025, Table 3). Lower jaw displacement had a negative trend with cranium length that was non-significant (Figure 5C, Table 3). As cranium length increased, the angular displacement of the lower jaw decreased, however, not at a rate predicted by GSM, with the *t*-test revealing the observed least squares slope being significantly different from a GSM slope of 0 (Table 3).

**Table 3:** Scaling relationships for the eight kinematics variables measured from the feeding events of 36 individuals. *p*-values were generated from the *t*-tests comparing observed slopes with those expected under the GSM. Asterisks (*) next to *p*-values denote significance at the appropriate Bonferroni corrected values.

				*Linear Regression*				*t-test*	
Variable	Average (mean ± SE)	Expected GSM Slope	Observed Least Square Slope	y-Intercept	*r^2^*	*F*	*p-value*	*t*	*p-value*
log Cranium Length (cm)	1.18 ± 0.147	1	0.970	−0.830	0.903	37.065	0.004*	−0.5988	>0.500
log Lower Jaw Length (cm)	1.23 ± 0.159	1	1.050	−0.940	0.910	40.652	0.003*	0.9198	>0.200
log Upper Jaw Length (cm)	0.78 ± 0.146	1	0.970	−1.220	0.917	44.383	0.003*	−0.4799	>0.500
log Lower Jaw Width (cm)	0.74 ± 0.158	1	1.010	−1.360	0.955	23.656	0.008*	0.1935	>0.500
log Maximum Gape (mm)	1.44 ± 0.049	1	0.589	0.332	0.071	2.599	0.116	−16.023	<0.001*
log Cranial Rotation (degree)	1.06 ± 0.078	0	0.498	0.119	0.020	0.696	0.410	11.964	<0.0025*
log Lower Jaw Displacement (degree)	1.40 ± 0.065	0	−0.581	2.500	0.040	1.405	0.244	−14.225	<0.001*
log Maximum Gape Velocity (mm·s^−1^)	2.29 ± 0.082	0	−0.015	2.320	2.000 × 10^−5^	0.001	0.981	−0.3068	>0.250
log Cranial Rotation									
Velocity (degrees·s^−1^)	0.67 ± 0.077	−1	−0.956	2.469	0.076	2.799	0.103	0.969	>0.250
log Lower Jaw Displacement									
Velocity (degree·s^−1^)	0.50 ± 0.088	−1	−0.491	1.426	0.016	0.538	0.468	10.495	<0.005*
log Time to Maximum Gape (s)	0.21 ± 0.039	1	0.340	-0.427	0.037	1.319	0.259	42.357	<0.001*
log Length of Feeding Bout (s)	0.34 ± 0.052	1	−0.127	0.580	0.003	0.102	0.752	−40.078	<0.001*

The velocity measurements were highly variable across cranium lengths (Figure 5E–G). Maximum gape velocity had no relationship to cranial length as indicated by the slope of −0.015 and the very low coefficient of determination (*r*^2^ = 2.000 × 10^−5^; Table 3). However, when comparing the observed least squares slope for maximum gape velocity with the predicted slope from the GSM, the *t*-test revealed that there was no significant difference between observed and predicted, indicating that the observed trend followed the model prediction, mainly that maximum gape velocity remains constant with increasing cranium length (*p*-value > 0.250, Table 2). Both cranial rotation and lower jaw displacement velocities had negative, non-significant trends with cranium length. As the cranium length increased the velocity at which the maximal angle was displaced slowed (Figure 5F,G, Table 3). Both of these velocity measurements had large variability in the data as indicated by the very low coefficients of determination (ranging in *r*^2^ from 0.016 to 0.076). Results of the *t*-tests, indicated no significant difference between the observed least squares slope for cranial rotation velocity and the slope predicted by the GSM (*p*-value > 0.250), yet there was a significant difference between the observed slope of the lower jaw displacement velocity and the slope predicted from the GSM (*p*-value < 0.005) (Table 3).

For both timing variables the coefficients of determination were very low (*r*^2^_time maximum gape_ = 0.037; *r*^2^_feeding bout_ = 0.003) indicating no relationship between cranium size and timing events and that timing events remained constant throughout this size range (Figure 5D,H, Table 3). Time to maximum gape had a slight, positive trend with cranium length, whereas the duration of the feeding bout had a slight decrease as the cranium length increased. Results of the *t*-tests revealed that the observed slopes for both timing variables were significantly different from the slopes predicted by GSM (*p*-values < 0.001, Table 3).

This study sought to describe the scaling relationships of linear, angular and timing excursions in young *A. mississippiensis* during prey-capture events in light of the isometric scaling of the cranial elements. Even though this study indicated that the log-transformed skeletal jaw elements scaled isometrically with log-transformed body size (Figure 4, Table 3), both maximum gape and cranial rotation had slight positive trends, and were significantly different from the predictions of the GSM (in isometry for maximum gape [slope =1] and a slope = 0 for cranial rotation). The trends generated indicate that as individuals grew their maximum gape and degree of cranial rotation increased. Ontogenetically, *A. mississippiensis* undergoes changes in its feeding morphology [3,4], from short and broad snouts to elongate and slender snouts, which may explain the trends seen above. Juvenile alligators tend to have a diet composed of fish and invertebrates, putting a premium on speed of closing the jaws to capture very mobile prey, to the detriment of bite-force production [4,5,11,12,13]. On the contrary, adults tend to have a diet composed of small mammals and turtles, of which require tremendous mechanical force for crushing, with reduced speed [4,5,13]. Studies have indicated that the m. adductor mandibulae externus profundus (MAMEP) and m. pterygoideus anterior and posterior (MPt) are vital in jaw closure and change throughout ontogeny; with increasing number of parallel fibers and fiber length as the individual grows [3,4,22]. This trade-off in speed and power are invariably linked to the alligator’s shift in diet. When investigating the relationship between length and tension on resting sarcomeres, the cranial muscle lengths would be influenced by rotation of the cranium and by gape. Perhaps this hypoallometric relationship between this linear/angular excursion and size is the consequence of optimal functioning of these jaw closing muscles.

Interestingly, when investigating the relationship between maximum gape velocity and the timing variables, slopes were close to zero, demonstrating that those variables are constant through ontogeny. Although this is interpreted with caution, as this part of the study was limited to two groups of animals close in size/age (hatchlings and juveniles). Possible differences in large juveniles, sub-adults and adults are an area for future study. The force generated by a muscle is a function of its velocity. Here, results illustrate conformity from hatching to juvenile life stages in the alligator’s ability to open its mouth and time to complete the prey-capture event. The consistency of timing variables across size may indicate that a premium is put on force production, with increasing muscle recruitment, rather than velocity and timing of the feeding event as the alligator grows [4,9]. With the cranial elements undergoing isometric growth, increasing in size and weight, more muscle mass and fiber recruitment would be required to maintain movement of the elements at a constant rate through ontogeny [3,4,22].

An allometric relationship has been established between bite-force performance and head length, with force increasing with size in proportions not predicted from isometric growth (slope = 2.75 compared with an isometric slope of 2.00) [4]. It was hypothesized that this relationship may be due to the unfused nature of the juvenile skeletal elements that undergo considerable flexion between the balancing side relative to the working side during the bite-force trials. This, in turn, may affect the transfer of force from the muscles to the bite point and result in lower biting forces [4]. Four of the eight kinematic variables had increasing trends with cranium length, indicating that alligator prey capture function may follow the allometric relationship in bite-force performance [4], however, larger individuals need to be added to establish a more precise relationship. Lower jaw displacement, lower jaw displacement velocity and cranial rotation velocity share negative trends with cranium length (Table 3). This indicates that as the individual grows there is a reduction in these kinematic variables. These relationships could be the result of shifts between speed and power through ontogeny as well. It has also been hypothesized that the bite-force allometry could be a result of a trade-off in morphology, with a shift away from sharp and slender dentition (good for increased velocity and grasping prey) to more blunt and robust dentition that requires more force to penetrate the prey [4,9].

High variability in the data led to very small r^2^ values, making it difficult to determine the definitive relationship between cranium length and the kinematic variables measured. However, two variables emerge, maximum gape and lower jaw displacement, which have reduced variability in their data compared with the other variables measured (Figure 5A,C). The alligator’s ability to increase its lower jaw displacement influences its overall maximum gape, and in turn, the size of the prey item it can manipulate. Intriguing is the reduction of the lower jaw displacement with size, although the maximum gape increases with size. Although there is high variability, there is a slight positive trend in increasing cranial rotation with size, in effect making up the difference in reduction in lower jaw displacement to the overall increase in maximum gape with size.

## 4. Conclusions

Although it has been shown that there is an isometric relationship between cranial elements and body size in juvenile *A. mississippiensis*, these isometric relationships are not transferred to linear and timing variables of prey-capture events in these two size classes studied. These trends echo results of other investigations of scaling relationships examining bite-force production [4,9] and terrestrial locomotion [3,5,6,7]. A study investigating the aquatic feeding behavior and kinematics of *Salamandra salamandra* just after birth and 59 days later indicated that the morphometrics of the cranial elements across the two ontogenetic stages scaled isometrically with snout-vent length, maintaining geometric similarity over that size range [23]. Nevertheless, there was a lack of kinematic change through *S. salmandra* ontogeny indicating that feeding kinematics do not scale with body size, contrary to model predictions [23]. Another study inspecting scaling of the feeding kinematics in *Ginglymostoma cirratum* (nurse shark) indicated that while the feeding apparatus grows isometrically, its function may not change ontogenetically [24].

An interesting aspect of *A. mississippiensis* feeding behavior that was not examined is the influence of an aquatic environment on the scaling relationships investigated here. Other researchers have called attention to the fact that water is 700 times more dense than air, and that this physical property alone would modify the ability and behavior of prey capture in any organism in an aquatic environment [2,25]. Based on the physical properties of water compared with air and the feeding behaviors articulated in previous research, prey capture is bound to be more dynamic and intricate than that presented here [25]. Foraging behavior and activity patterns are another factor that would play a huge role in successful prey capture. It has been documented that prey-capture success in *A. mississippiensis* is highest in the morning, becoming quite reduced by sunset [25]. It was also observed that an individual’s position in the water column significantly affects success, with greater success achieved when attacking prey while submerged [26].
